# Electrochemical Immunosensor for the Simultaneous Determination of Two Main Peanut Allergenic Proteins (Ara h 1 and Ara h 6) in Food Matrices

**DOI:** 10.3390/foods10081718

**Published:** 2021-07-25

**Authors:** Maria Freitas, Marta M. P. S. Neves, Henri P. A. Nouws, Cristina Delerue-Matos

**Affiliations:** 1 REQUIMTE/LAQV, Instituto Superior de Engenharia do Porto, Instituto Politécnico do Porto, Rua Dr. António Bernardino de Almeida 431, 4200-072 Porto, Portugal; maria.freitas@graq.isep.ipp.pt (M.F.); marta.pereira-da-silva-neves@warwick.ac.uk (M.M.P.S.N.); cmm@isep.ipp.pt (C.D.-M.); 2 Department of Chemistry, Institute of Advanced Study, University of Warwick, Coventry CV4 7AL, UK

**Keywords:** Ara h 1, Ara h 6, electrochemical dual sensor, food allergy, peanut allergen, simultaneous analysis

## Abstract

Efficiently detecting peanut traces in food products can prevent severe allergic reactions and serious health implications. This work presents the development of an electrochemical dual immunosensor for the simultaneous analysis of two major peanut allergens, Ara h 1 and Ara h 6, in food matrices. A sandwich immunoassay was performed on a dual working screen-printed carbon electrode using monoclonal antibodies. The antibody–antigen interaction was detected by linear sweep voltammetry through the oxidation of enzymatically deposited silver, which was formed by using detection antibodies labeled with alkaline phosphatase and a 3-indoxyl phosphate/silver nitrate mixture as the enzymatic substrate. The assay time was 2 h 20 min, with a hands-on time of 30 min, and precise results and low limits of detection were obtained (Ara h 1: 5.2 ng·mL^−1^; Ara h 6: 0.017 ng·mL^−1^). The selectivity of the method was confirmed through the analysis of other food allergens and ingredients (e.g., hazelnut, soybean and lupin). The dual sensor was successfully applied to the analysis of several food products and was able to quantify the presence of peanuts down to 0.05% (*w*/*w*). The accuracy of the results was confirmed through recovery studies and by comparison with an enzyme-linked immunosorbent assay. Tracking food allergens is of utmost importance and can be performed using the present biosensor in a suitable and practical way.

## 1. Introduction

The diversity of dietary patterns in human nutrition and the widespread incidence of food intolerances, allergies and hypersensitivity have led to changes in eating habits. Food allergy involves an adverse response of the immune system to the presence of specific allergens, and the most usual symptoms include cutaneous, respiratory, gastrointestinal and cardiovascular complications [1]. Individuals that suffer from food allergies require accurate restrictive measures (i.e., avoiding allergen-containing foods) and personalized treatments [2,3].

Peanuts (*Arachis hypogaea*) are recognized as one of the most common allergy-causing foods, and therefore their presence needs to be clearly indicated in the list of dietary allergens (EC (2011) Regulation (EU) No 1169/2011) [4]. However, despite its high allergenic impact, the peanut is also an important legume crop and a functional food with increasing use, especially in healthy food products [5]. Moreover, due to its nutritional value and status as a source of plant-based proteins, the peanut is associated with health benefits [6,7]. To date, the major peanut protein allergens (Ara h) have been identified and categorized into a select group of structural protein superfamilies [8]: the cupin superfamily (Ara h 1 and Ara h 3) and the prolamin superfamily (Ara h 2) [8,9]. In susceptible individuals, the exposure to these allergens can lead to allergic episodes, normally mediated by immunoglobulin E, and the resulting symptoms can range from mild to severe, such as anaphylaxis, a potentially life-threatening reaction that requires immediate emergency treatment [1]. Recent studies suggest that Ara h 6, which belongs to the prolamin family and shares a high similarity in sequence and structure with Ara h 2, also presents a serious risk to sensitized individuals and should be considered a major allergen [10,11]. More specifically, a mono-sensitization to Ara h 6, despite being unusual, can cause severe allergic reactions, such as anaphylaxis, even in the absence of sensitization to Ara h 1 or Ara h 2 [12].

Therefore, effective labeling of commercial products can prevent unnecessary exposure to allergen-containing foods, avoiding the consequent health implications. Indeed, strict requirements for labeling commercial food products containing traces of a specific allergen or even possible allergenic ingredients were imposed by European directives and the Food Allergen Labeling and Consumer Protection Act (FALCPA) of the U.S. Food and Drug Administration (EC (2003) Directive 2003/89/EC; FALCPA 2004, Public Law 108–282, Title II) [13,14,15]. This has led to several challenges including the analysis of food allergens (required for consumer protection and/or food fraud identification), efficient product labeling and food manufacturers’ compliance with legislation.

Most standardized methods and research tools for the analysis of peanut-containing food are based on molecular biology, immunoassays and chromatographic techniques [16]. These methods are laborious and time-consuming and require extensive expertise. Therefore, to simplify these analyses, the use of biosensors as high-performance analytical tools for tracking peanut-containing food products has increased, especially for the rapid detection of DNA and/or proteins. These decentralized devices can compete with the traditional techniques and contribute to the reduction in the risk of accidental ingestion and, thus, to avoiding affecting allergy-prone individuals [17]. Advances in the biosensing field have been reported, and electrochemical biosensors appear as an outstanding methodology for allergenic protein analysis [18]. Moreover, specific analysis of trace amounts of allergens related to processing/cross-contamination, or even adulterants and hidden substances of by-products used in the manufacture of emerging functional foods, can be accomplished efficiently using immunosensors in a sustainable and user-friendly way [19,20]. Furthermore, multiplex in situ detection using biosensors can considerably improve the testing capacity in a sustainable way by reducing the sample volume and quantity of tests and avoiding expensive equipment.

Based on a simultaneous analysis approach, the present work reports, for the first time, an electrochemical dual immunosensor for the determination of the peanut allergens Ara h 1 and Ara h 6. Dual-SPCEs were used, and an enzymatic label (alkaline phosphatase) combined with a specific substrate (3-indoxyl phosphate and silver nitrate) was employed to obtain the electrochemical signal through the oxidation of metallic silver by linear sweep voltammetry. The target analytes were quantified in 2 h 20 min. Due to the specific recognition of the allergens, using monoclonal capture and detection antibodies, and the simplicity of the detection method, the present methodology was recently successfully applied in our group for the simultaneous analysis of biomarkers in clinical analysis [21]. However, its application to food control has not yet been reported. A set of food products were tested and analyzed using the sensor, and conventional ELISA kits were used for method validation.

## 2. Materials and Methods

### 2.1. Equipment

Electrochemical measurements were performed using a µStat 200 bipotentiostat controlled by DropView software (DropSens, Oviedo, Spain). A DRP-BICAST connector was used to interface the bipotentiostat and the customized dual screen-printed carbon electrodes (dual-SPCE, DRP-X1110, DropSens, incorporating two carbon elliptic-shaped working electrodes (WE, A = 0.063 cm^2^), a carbon auxiliary electrode and a silver pseudo-reference electrode).

For sample preparation, a block thermostat (Tembloc, Selecta, Barcelona, Spain) and two centrifuges (Heraues Megafuge 16R and Heraeus-Fresco 21, Thermo Fisher Scientific, Osterode am Harz, Germany) were used.

The evaluation of the accuracy of the assay’s results was performed using ELISA kits. For these analyses, a multi-mode microplate reader (Synergy HT W/TRF, BioTek Instruments, Winooski, VT, USA) was used, and the data were treated with Gen5 Version 2.0 data analysis software (BioTek Instruments, Winooski, VT, USA).

### 2.2. Reagents and Solutions

Ara h 1 standard, monoclonal antibody anti-Ara h 1, mAb IgG1, 2C12 (Capture antibody—CAb), biotinylated monoclonal antibody anti-Ara h 1, mAb IgG1, 2F7 (Detection antibody—DAb), Ara h 6 standard, monoclonal antibody anti-Ara h 6, mAb IgG1, 3B8 (Capture antibody—CAb), biotinylated monoclonal antibody anti-Ara h 6, mAb IgG1, 3E12 (Detection antibody—DAb), the respective ELISA kit (Cat. No.: 2C12/AH1 (Ara h 1), 3B8/3E12 (Ara h 6)) and Ara h 2 standard were purchased from Indoor Biotechnologies. Bovine serum albumin fraction V (BSA), casein sodium salt from bovine milk, silver nitrate, 3-indoxyl phosphate (3-IP), tris(hydroxymethyl)aminoethane (Tris) and nitric acid (≥65%) were purchased from Sigma-Aldrich. Streptavidin–alkaline phosphatase conjugate (STR–ALP) was obtained from Thermo Fisher Scientific.

Working solutions of casein, BSA and the immunoreagents (Ara h 1, Ara h 6, CAb and DAb) were prepared in 0.1 M Tris-HNO_3_ pH 7.2 (buffer 1). Working solutions of STR–ALP were prepared in 0.1 M Tris-HNO_3_ pH 7.2 containing 2 mM Mg(NO_3_)_2_ (buffer 2). The substrate mixture (3-IP/Ag^+^) was prepared in 0.1 M Tris-HNO_3_ pH 9.8 containing 20 mM Mg(NO_3_)_2_ (buffer 3) and stored protected from light at 2–8 °C until use. All these solutions were prepared daily, using ultra-pure water (Simplicity 185, Millipore).

### 2.3. Immunoassay Strategy

**Figure 1:**
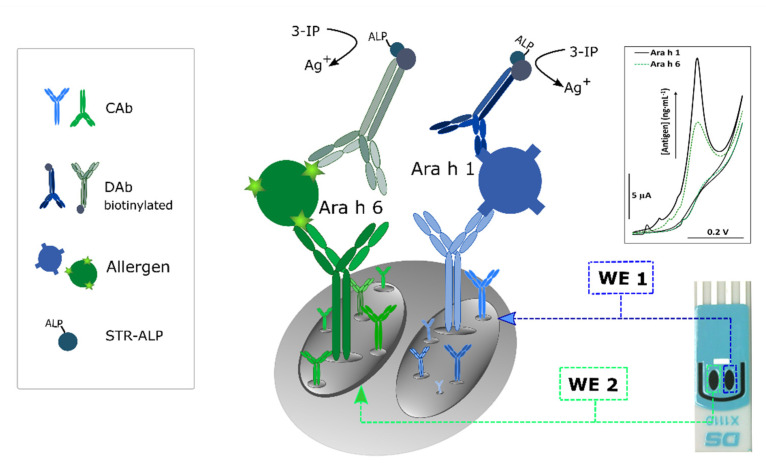
Schematic illustration of the immunosensor’s configuration and construction.

Figure 1 illustrates the single-use assay procedure. In summary, the optimized assay consisted of:

(i) Modification of the working electrodes (WE 1 and WE 2) of the dual-SPCE with the CAbs (5 μL, 25 μg·mL^−1^), anti-Ara h 1 and anti-Ara h 6, respectively, overnight at 4 °C. Physical adsorption was selected as the immobilization method of the CAbs on the electrode surface due to its simplicity and cost-effectiveness;
(ii) After washing with buffer 1 to remove unbound CAb, the free surface sites were blocked with casein (50 μL, 2% (*w*/*v*), 30 min);
(iii) After washing with buffer 1, a mixed standard solution (containing Ara h 1 and Ara h 6) or a diluted sample extract (1000-times dilution) (30 μL, 60 min) was placed on the sensing surface;
(iv) After washing with buffer 1, a mixture of the DAbs (250-times dilutions) and BSA (1% *w*/*v*) (30 μL, 30 min) was placed on the dual-SPCE;
(v) After washing with buffer 2 to remove unbound DAb, a 30 μL aliquot of the enzymatic substrate (STR–ALP; 1:150,000) containing BSA (0.1% *w*/*v*) was added for 30 min, which was followed by a final washing step with buffer 3;
(vi) Once the affinity events were completed, the enzymatic reaction took place (20 min) by using a mixture (80 μL) of a solution containing 3-IP (1.0 mM) and silver nitrate (0.4 mM) that covered the electrochemical cell.

The detection mechanism used in this work consisted of the electrochemical oxidation of enzymatically deposited silver; ALP converts 3-IP in an unstable indoxyl intermediate whose oxidation leads to the reduction of silver ions into metallic silver, which will be co-deposited with indigo blue. Finally, the enzymatically deposited metal can be detected through stripping analysis [22]. In this work, a linear sweep voltammogram was recorded from 0.0 to + 0.35 V at a scan rate of 50 mV s^−1^.

### 2.4. Sample Preparation

Raw peanuts (unknown variety), powdered peanut butter, an allergen-free vegan cookie, a cookie containing 8% of peanut (peanut cookie), a cookie that may contain traces of peanut and other nuts (normal cookie), a snack containing dried apple and peanuts (healthy snack), oat, pineapple, sesame, hazelnut, lupin, soybean and buckwheat flour were purchased from local supermarkets and prepared according to a previously described protocol [23], with slight modifications. Briefly, samples were ground and homogenized in a grinder (3×, 20 s), and 1 g of the ground sample was mixed with 10 mL of extraction buffer (0.1 M Tris-HNO_3_, pH 8.2) and stirred continuously at 60 °C for 30 min. This mixture was then centrifuged for 5 min at 5000 rpm, and 1 mL of the aqueous phase was collected and centrifuged (4 °C) for an additional 5 min at 10,000 rpm. An aliquot was then diluted (1000×), and 30 μL was used to perform the assay.

The immunoassay was used to analyze foods containing unknown amounts of peanut, peanut-containing foods, legumes and allergen-free products from organic farming.

## 3. Results and Discussion

### 3.1. Optimization of the Immunosensor

One of the main issues when using sensors with multiple working electrodes is the possible crosstalk between them. The results obtained with the dual sensor were compared with the ones obtained with a dual-SPCE, with both WE1 and WE2 only detecting Ara h 1 or Ara h 6 (‘no dual sensor’) (Figure S1A,B). The results obtained (Figure S1C) corroborated that no crosstalking occurred between both working electrodes.

To study the blocking of non-occupied sites on the transducer surface, BSA (2% (*w*/*v*)) and casein (2% (*w*/*v*)) solutions were used. The signal-to-blank (S/B) ratio in the presence and absence of the analytes was used to select the optimum values. Compared to BSA, casein was able to efficiently minimize non-specific adsorption, achieving the best S/B ratio (Figure S2). This is probably related to the lower molecular weight proteins (19–25 kDa) in casein compared to BSA (66.5 kDa) that, due to their higher surface-to-volume ratio, can improve the blocking of non-specific adsorptions of other proteins on the electrode surface [24]. Accordingly, casein was selected as the blocking agent.

The non-specific adsorption of the target proteins and the capture and detection antibodies were evaluated to confirm the correct performance of the proposed sandwich immunoassay (Figure S3). For this purpose, control assays using a blank solution (A) and a solution containing 250 ng·mL^−1^ Ara h 1 and 2.5 ng·mL^−1^ Ara h 6 (B) were performed. The peak current intensities (*i*_p_) obtained in assays without Ara h 1 CAb (C), Ara h 6 CAb (D), Ara h 1 DAb (E), Ara h 6 DAb (F) or both DAbs (G) were compared with the control assays. As it can be observed in Figure S3, for all these cases, the obtained *i*_p_ was similar to the blank signal, resulting from residual non-specific protein binding. The influence of the enzymatic substrate components on the electrochemical signal was also studied. As it was expected, in the assays in which the enzyme, the enzymatic substrate or Ag^+^ was not used, no peaks were observed. These results corroborate the appropriate recognition events between the analyte and both the capture and the detection antibodies as well as the effectiveness of the blocking step. The influence of the temperature was also studied (25 and 30 °C), and Figure S4 shows that at 30 °C, higher *i*_p_ values were obtained.

**Figure 2:**
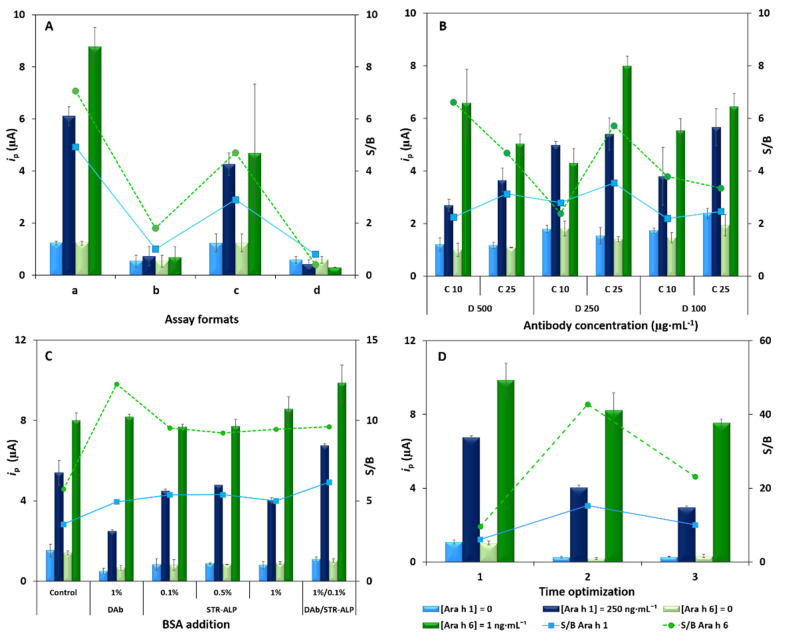
Results of the optimization. (**A**) Different assay strategies: a—4 incubation steps (1—blocker, 2—target analytes, 3—DAb, 4—enzymatic label), b—3 incubation steps (1—blocker, 2—target analytes, 3—mixture of DAb and enzymatic label), c—3 incubation steps (1—blocker, 2—mixture of target analytes and DAb, 3—enzymatic label) and d—2 incubation steps (1—blocker, 2—mixture of target analytes, DAb and enzymatic label). (**B**) Study of the concentration of CAb (10 and 25 µg·mL^−1^) and DAb (dilution factor: 100, 250 and 500). (**C**) Effect of the addition of BSA 1% (*w*/*v*) to Dab, and BSA 0.1, 0.5 and 1% (*w*/*v*) to STR–ALP. (**D**) Influence of the incubation times: 1—control assay—allergens 60 min and DAb 60 min, 2—allergens 60 min and DAb 30 min, 3—allergens 30 min and DAb 60 min (Ara h 1: 0 (blank) and 250 ng·mL^−1^; Ara h 6: 0 (blank) and 1 ng·mL^−1^; error bars correspond to the standard deviation of 3 replicates).

After these studies, the assay format was evaluated: (a) four incubation steps (1—blocker, 2—target analytes, 3—DAb, 4—enzymatic label), (b) three incubation steps (1—blocker, 2—target analytes, 3—mixture of DAb and enzymatic label), (c) three incubation steps (1—blocker, 2—mixture of target analytes and DAb, 3—enzymatic label) and (d) two incubation steps (1—blocker, 2—mixture of target analytes, DAb and enzymatic label). In these assays, a blank and a solution containing 250 ng·mL^−1^ Ara h 1 and 1 ng·mL^−1^ Ara h 6 were tested. As it can be observed in (Figure 2A), format (a) provided the best S/B ratios, with good precision. Therefore, format (a) was selected to continue the development of the immunoassay.

The reagents’ concentrations and incubation/reaction times were also tested. Two different CAb concentrations and three different DAb dilutions, in a total of six combinations per analyte, were tested. A blank and a solution containing 250 ng·mL^−1^ Ara h 1 and 1 ng·mL^−1^ Ara h 6 were used. For both analytes, and as a compromise between the S/B ratio and precision, a CAb concentration of 25 µg·mL^−1^ combined with a DAb solution diluted 250 times yielded the best performance (Figure 2B). Afterwards, BSA (1% (*w*/*v*)) was added to the DAb solution and to the STR–ALP solution (0.1, 0.5 and 1% (*w*/*v*)) to evaluate the possibility of decreasing the blank signal without compromising the S/B ratio. As it can be observed in Figure 2C, the addition of BSA (1% (*w*/*v*)) to the DAb solution, and BSA (0.1% (*w*/*v*)) to the STR–ALP solution provided better overall results for both analytes. In addition, to try to reduce the assay time, shorter incubation times of the allergens and the DAb were tested and compared (Figure 2D): (1) control assay—allergens 60 min and DAb 60 min, (2) allergens 60 min and DAb 30 min, (3) allergens 30 min and DAb 60 min. The incubation of the analytes for 60 min and the Dab for 30 min provided the best S/B ratio. Table S1 summarizes the optimized experimental variables and the selected values.

### 3.2. Analytical Characteristics

**Figure 3:**
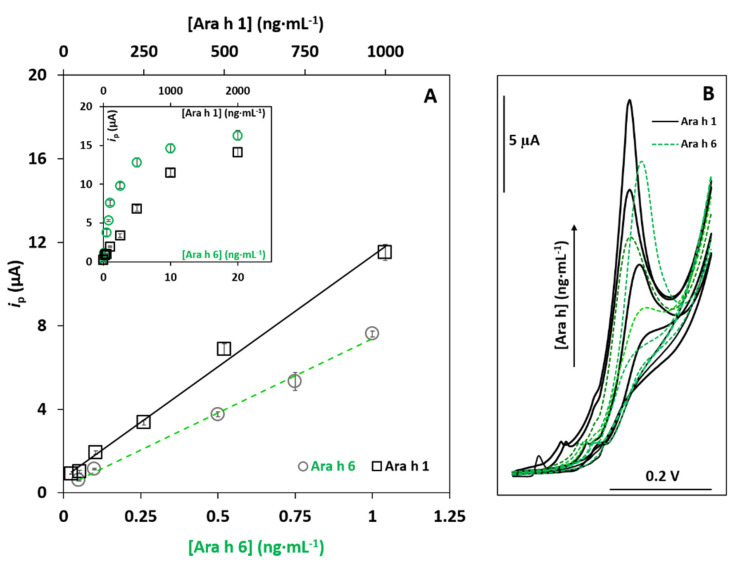
(**A**) Calibration straights using the dual-SPCE for Ara h 1 and Ara h 6 analysis. ((Ara h 1) (ng·mL^−1^): 0, 25, 50, 100, 250, 500 and 1000; (Ara h 6) (ng·mL^−1^): 0, 0.050, 0.10, 0.50, 0.75 and 1.0). Inset: plots including all the tested concentrations (averages and standard deviations are shown, *n* = 4). (**B**) LSV voltammograms recorded within the linear range for Ara h 1 (solid line) and Ara h 6 (dashed line).

The analytical responses of the immunosensor to different concentrations of the allergens were evaluated (between 25 and 2000 ng·mL^−1^ for Ara h 1, and between 0.05 and 20 ng·mL^−1^ for Ara h 6), and a linear relationship between *i*_p_ and the protein concentration was found between 25 and 1000 ng·mL^−1^ (six concentrations) for Ara h 1 (*i*_p_ (µA) = 0.0111 ± 0.0004 (Ara h 1) (ng·mL^−1^) + 0.72 ± 0.21; r = 0.997), with a sensitivity of 0.175 µA·mL·ng^−1^·cm^−2^, and between 0.050 and 1.0 ng·mL^−1^ (five concentrations) for Ara h 6 (*i*_p_ (µA) = 7.10 ± 0.28 (Ara h 6) (ng·mL^−1^) + 0.28 ± 0.17; r = 0.998), with a sensitivity of 113 µA·mL·ng^−1^·cm^−2^. The corresponding calibration plots are presented in Figure 3A, the respective LSV voltammograms are presented in Figure 3B and Table S2 summarizes the figures of merit for both allergens.

The limits of detection (LOD) and quantification (LOQ) were, respectively, calculated from the calibration plot according to the 3 *s*_b_/*m* and 10 *s*_b_/*m* equations, where *s*_b_ is the standard deviation of the blank signal, and *m* is the slope of the calibration plot. For Ara h 1, an LOD of 5.2 ng·mL^−1^ and an LOQ of 17 ng·mL^−1^ were obtained, while for Ara h 6, the obtained values were 0.017 ng·mL^−1^ and 0.055 ng·mL^−1^, respectively. The coefficient of variation of the method (V_x0_) demonstrated the adequate precision of the method since V_x0_ ≈ 10% was obtained for Ara h 1 and 3.3% was obtained for Ara h 6.

The precision of the slope of the calibration straight was evaluated using three plots determined on different days. The relative standard deviations (RSD) were 9.4% and 1.4% for Ara h 1 and Ara h 6, respectively. The obtained values were both acceptable; however, they demonstrated that the sensor was more precise for Ara h 6 than for Ara h 1.

**Figure 4:**
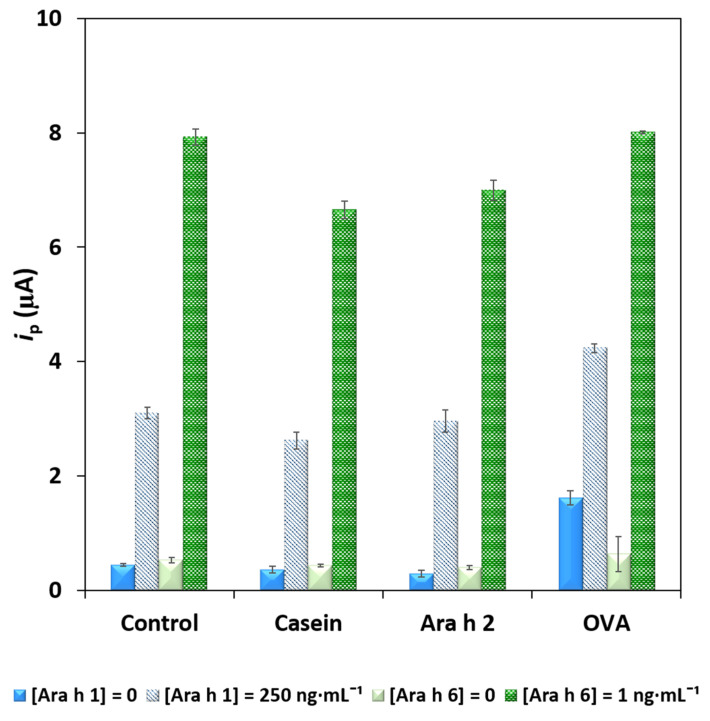
Evaluation of the selectivity (non-target proteins: casein (3%), Ara h 2 (250 ng·mL^−1^) and OVA (130 mg·mL^−1^)) and interference studies using Ara h 1 (0 (blank) and 250 ng·mL^−1^) and Ara h 6 (0 (blank) and 1 ng·mL^−1^). Error bars correspond to the standard deviation of 3 replicates.

To evaluate possible interferences and the selectivity of the sensor, other food components and protein allergens were tested. Ara h 2 was chosen because it is another peanut allergen, and casein (from bovine milk) and albumin (from chicken egg white, OVA) were selected because some of the product labels indicated that milk and egg are, or could be, present. The dual sensor’s response was studied by adding Ara h 2 (250 ng·mL^−1^), casein (3%) and OVA (130 mg·mL^−1^) to the blank and solutions containing Ara h 1 (250 ng·mL^−1^) and Ara h 6 (1 ng·mL^−1^). The obtained *i*_p_ values are presented in Figure 4. The presence of the tested non-target proteins did not result in significant differences, except for the blank signals in the presence of Ara h 2 and OVA, which were irreproducible and slightly higher, although without overlapping the *i*_p_ values obtained in the presence of Ara h 1 or Ara h 6. Importantly, although Ara h 2 and Ara h 6 are structurally similar [25], no significant cross-reactivity was observed, confirming the dual sensor´s selectivity towards Ara h 1 and Ara h 6.

The sensor’s storage stability was assessed over a 60-day period (Figure S5). The as-prepared dual sensor was stored at 4 °C, and no significant differences in *i*_p_ values were observed for up to 30 days, obtaining 103.7% of the initial signal for Ara h 1, and 101.1% for Ara h 6. After 60 days, a reduced signal for both allergens was noticeable (retaining 42.1% of the initial signal for Ara h 1, and 45.5% for Ara h 6).

### 3.3. Sample Analysis

Due to the absence of a certified reference material, recovery studies were performed to assess the accuracy of the sensor’s results in the analysis of food samples. For this purpose, an allergen-free product (vegan cookie) was selected to quantify the peanut allergens at trace levels using aliquots of the same sample (1 g), prepared simultaneously, and analyzed before and after being spiked with a known amount of allergen (Ara h 1: 0, 50, 100, 500 and 1000 ng·mL^−1^; Ara h 6: 0, 0.050, 0.10, 0.50 and 1.0 ng·mL^−1^). Recoveries for Ara h 1 varied between 87% and 115%, and between 91% and 116% for Ara h 6. This proves that the sensor provided accurate results.

The sensor’s ability and usefulness for the analysis of the target proteins in commercial food matrices were tested. Raw peanuts (unknown variety) were analyzed to quantify Ara h 1 (6.1 ± 0.8 mg·g^−1^) and Ara h 6 (18.1 ± 0.6 µg·g^−1^). The allergen-free vegan cookies were spiked with increasing amounts of peanuts: 0.05%, 0.1%, 0.5%, 1% and 8% (*w*/*w*). Additionally, peanut cookies whose label indicates the presence of 8% of peanut in their composition were also tested. The results obtained for the 8% spike and the peanut cookie were in close agreement (Figure S6). As it can be seen in Figure S6, the sensor was able to detect peanuts at amounts as low as 0.05%. Considering that current legislation requires a declaration of the presence of peanuts (either as a peanut-containing ingredient or possible cross-contamination in the manufacturing process), and that the minimal clinical known amount that may cause the slightest effect in sensitized individuals is less than 0.1% (*w*/*w*) of peanut, it can be inferred that the present sensor allows an effective determination of the allergens under study [1].

A set of commercial food samples (with and without the target allergens) was purchased in a local supermarket and analyzed: powdered peanut butter; an allergen-free vegan cookie; a cookie that may contain traces of peanuts and other nuts (normal cookie); a snack containing dried apple and peanuts (healthy snack); a cookie containing 8% of peanut (peanut cookie). Additionally, ingredients listed on the products’ labels (buckwheat flour, oat, pineapple, sesame, soybean) were analyzed to test matrix effects and possible cross-reactivities (Table 1). The presence of peanuts was only detected in the powdered peanut butter (3.95 mg·g^−1^ Ara h 1, and 11.1 µg·g^−1^ Ara h 6), the peanut cookie (1.37 mg·g^−1^ Ara h 1, and 12.9 µg·g^−1^ Ara h 6) and the healthy snack (2.83 mg·g^−1^ Ara h 1, and 12.0 µg·g^−1^ Ara h 6). The analysis of the individual ingredients once again showed the selectivity of the sensor.

Furthermore, the samples were also analyzed using commercial ELISA kits (Table 1 and Figure S7). The good correlations, and slopes near to 1, between the results for both allergens confirmed the accuracy of the sensor’s results. Although immunoassays (e.g., ELISA) are widely available, they involve costly kits and require considerable assay times, as well as trained personnel and expensive equipment. Additionally, the LODs of the ELISA kits are higher than the ones obtained with the developed immunosensor.

### 3.4. Comparison with Other Electrochemical Immunoassays for the Analysis of Ara h 1 and Ara h 6

Biosensors have practical utility and enable food product monitoring in real time. To date, only one work described a dual analysis strategy for the analysis of allergenic peanut proteins [26], and very few immunosensors have been described in the literature for the selective determination of Ara h 1 or Ara h 6 [27,28,29,30,31]. Table 2 presents a summary of the reported analytical characteristics of these assays. Some strategies for the single detection of individual allergens demonstrated shorter assay times, but with a time-consuming electrode surface modification and nanostructuration [27,29,30]. Additionally, the lack of applicability or stability studies is a shortcoming observed in the developed works.

The analytical characteristics of the developed immunosensor and its applicability to the simultaneous determination of Ara h 1 and Ara h 6 in a wide variety of commercial products do, in fact, prove its usefulness. Furthermore, these attractive characteristics reinforce the potential applicability of biosensors as portable platforms for multiple analyses of trace levels (or hidden) of protein allergens in food matrices.

## 4. Conclusions

The simultaneous quantification of two major allergenic peanut proteins (Ara h 1 and Ara h 6), based on a portable and miniaturized electrochemical dual-SPCE immunosensor, was reported for the first time. A set of analytical characteristics was studied, and the biosensor allows the analysis to be performed within 2 h 20 min, with a hands-on time of 30 min. The sensing surface was stable for 30 days. The low detection limits (Ara h 1: 5.2 ng·mL^−1^; Ara h 6: 0.017 ng·mL^−1^) enable food tracking and monitoring of trace levels of the target allergens. Selectivity and interference studies demonstrated that the matrix effects of the tested samples and the possible cross-reactions have a reduced impact on the results, and the presence of 0.05% (*w*/*w*) of peanut in the samples was successfully detected. The applicability to complex food matrices, and the validation of the results by comparison with ELISA kits and through recovery tests, allowed an effective proof of concept of the present dual immunosensor.

## Figures and Tables

**Table 1:** Quantification of Ara h 1 (mg·g^−1^) and Ara h 6 (µg·g^−1^) in a set of food products (dilution 1000×). Results of the developed electrochemical dual immunosensor and a commercial ELISA (spectrophotometric) kit (averages and standard deviations of 3 replicates).

Food/Ingredient	Ara h 1 (mg·g^−1^)		Ara h 6 (µg·g^−1^)	
	Dual Sensor	ELISA	Dual Sensor	ELISA
Heathy snack	2.83 ± 0.25	2.82 ± 0.09	12.0 ± 0.6	12.6 ± 0.07
Peanut cookie	1.37 ± 0.15	1.58 ± 0.07	12.9 ± 0.7	13.1 ± 0.08
Peanut butter	3.95 ± 0.18	3.96 ± 0.08	11.1 ± 0.7	12.1 ± 0.08
Vegan cookie	0.16 ± 0.06	0.11 ± 0.03	1.31 ± 0.12	1.01 ± 0.01
Normal cookie	0.21 ± 0.08	0.33 ± 0.02	0.60 ± 0.16	0.55 ± 0.04
Oat	0.20 ± 0.11	0.19 ± 0.08	1.22 ± 0.07	1.12 ± 0.03
Pineapple	0.056 ± 0.030	0.055 ± 0.011	1.49 ± 0.16	1.13 ± 0.12
Sesame	0.24 ± 0.09	0.20 ± 0.03	1.24 ± 0.29	1.01 ± 0.05
Hazelnut	0.028 ± 0.030	0.029 ± 0.011	2.22 ± 0.16	2.04 ± 0.12
Lupin	0.19 ± 0.03	0.15 ± 0.02	1.34 ± 0.29	1.28 ± 0.09
Soybean	0.029 ± 0.024	0.025 ± 0.020	2.20 ± 0.21	2.01 ± 0.18
Buckwheat flour	0.25 ± 0.09	0.17 ± 0.01	1.34 ± 0.30	0.91 ± 0.10

**Table 2:** Summary of analytical characteristics of electrochemical immunosensors for the detection of Ara h 1 and Ara h 6.

Allergen	Sensing Surface		Food Sample	Detection		LOD	Ref.
	Transducer (Preparation Time)	Stability		Technique	Assay Time		
Ara h 1, Ara h 6	Dual-SPCE (~12 h)	30 days	Lupin, buckwheat flour, sesame, soybean, vegan cookies, raw peanut, healthy snack, peanut butter, peanut cookies	LSV (3-IP/Ag^+^)	2 h 20 min	Ara h 1: 5.19 ng·mL^−1^; Ara h 6: 0.017 ng·mL^−1^	This work
Ara h 1	Silicon wafer/SWCNT(>15 h)	n.d.	n.d.	LSV (Label-free)	30 min	1 ng·mL^−1^	[27]
	SPCE/nAu (~12 h)	n.d.	Cookies, chocolate	LSV (3 IP/Ag^+^)	3 h 50 min	3.8 ng·mL^−1^	[28]
	AuE/11-MUA (~19 h)	n.d.	n.d.	EIS (Fe(CN)_6_^3−/4−^)	5 min	0.3 nM	[29]
Ara h 6	AuE/SWCNT (~14 h)	n.d.	Peanut butter, peanut pepper sauce, peanut chocolate, bean milk and chocolate milk	LSV (Label-free)	30 min	10 pg·L^−1^	[30]
	SPCE/nAu (~12 h)	n.d.	Cookies, chocolate	LSV (3-IP/Ag^+^)	3 h 50 min	0.27 ng·mL^−1^	[31]

3-IP—3-indoxyl phosphate; 11-MUA—11-mercaptoundecanoic acid; Ag^+^—silver ions; AuE—gold electrode; EIS—electrochemical impedance spectroscopy; H_2_O_2_—hydrogen peroxide; LSV—linear sweep voltammetry; nAu—nanostructured gold nanoparticles; SPCE—screen-printed carbon electrode; SWCNT—single-walled carbon nanotube. n.d.—no data.

## Data Availability

Not applicable.

## Supplementary Materials

The following are available online at https://www.mdpi.com/article/10.3390/foods10081718/s1, Figure S1: Schematic representation of the detection of Ara h 1 and Ara h 6 using a (A) No Dual sensor and (B) Dual sensor. (C) LSV voltammograms of the ‘No Dual’ and the Dual sensor (Ara h 1: 100·ng mL^−1^ and Ara h 6: 5 ng·mL^−1^), Figure S2: Study of the blocking agents BSA (2% (*w*/*v*)) and casein (2% (*w*/*v*)) and evaluation of non-specific adsorptions. (Ara h 1: 0 (blank) and 100 ng·mL^−1^; Ara h 6: 0 (blank) and 5 ng·mL^−1^; error bars correspond to the standard deviation of 3 replicates, Figure S3. Effect of non-specific adsorptions of the target proteins and the capture and detection antibodies. (A) Blank and (B) 250 ng·mL^−1^ Ara h 1 and 2.5 ng·mL^−1^ Ara h 6 (control assays), Assays without the use of: Ara h 1 CAb (C), Ara h 6 CAb (D), Ara h 1 DAb (E), Ara h 6 DAb (F), Ara h 1 DAb and Ara h 6 DAb (G). (Ara h 1: 250 ng mL^−^^1^; Ara h 6: 2.5 ng·mL^−^^1^), Figure S4. Current intensities obtained at 25 °C and 30 °C. (Ara h 1: 0 (blank) and 250 ng·mL^−1^; Ara h 6: 0 (blank) and 1 ng·mL^-1^; error bars correspond to the standard deviation of 3 replicates), Figure S5. Storage stability of the immunosensor (Ara h 1 (0 (blank) and 250 ng·mL^−1^) and Ara h 6 (0 (blank) and 1 ng·mL^−1^); error bars correspond to the standard deviation of 3 replicates), Figure S6. Quantification of Ara h 1 (mg·g^−1^) and Ara h 6 (mg·g^−1^) extracted from an allergen-free cookie sample spiked with increasing amounts of peanut and a peanut cookie, Figure S7. Correlation plots for the analysis of (A) Ara h 1 and (B) Ara h 6 in the tested food products using the developed immunosensor and ELISA kits. Error bars correspond to the standard deviation of 3 replicates, Table S1: Optimized experimental parameters, tested range, and selected values, Table S2. Figures of merit of the immunoassay.
