# Supplementary material for: Electrochemical Immunosensor for the Simultaneous Determination of Two Main Peanut Allergenic Proteins (Ara h 1 and Ara h 6) in Food Matrices
Source: Foods. 2021 Jul 25;10(8):1718. doi: 10.3390/foods10081718 (PMC8391283; doi:10.3390/foods10081718)
Supplement: Supplementary file 1 [file foods-10-01718-s001.zip › foods-1283800-supplementary.pdf]

Supplementary Material

Electrochemical Immunosensor for the Simultaneous Determination of Two Main Peanut Allergenic Proteins (Ara h 1 and Ara h 6) in Food Matrices

Maria Freitas [1,†], Marta M. P. S. Neves [1,2,†], Henri P. A. Nouws [1,*] and Cristina Delerue-Matos [1]

[1] REQUIMTE/LAQV, Instituto Superior de Engenharia do Porto, Instituto Politécnico do Porto, Rua Dr. António Bernardino de Almeida 431, 4200-072 Porto, Portugal; maria.freitas@graq.isep.ipp.pt (M.F.); marta.pereira-da-silva-neves@warwick.ac.uk (M.M.P.S.N.); cmm@isep.ipp.pt (C.D.-M.)

[2] Institute of Advanced Study, Department of Chemistry, University of Warwick, Coventry CV4 7AL, UK

* Correspondence: han@isep.ipp.pt

† Both authors contributed equally to this work.

Contents

Figure S1 .... S2

Figure S2 .... S3

Figure S3 .... S3

Figure S4 .... S4

Figure S5 .... S5

Figure S6 .... S5

Figure S7 .... S6

Table S1 .... S2

Table S2 .... S4

Table S1. Optimized experimental parameters, tested range, and selected values.

Experimental variable	Tested range	Selected value
[CAb] (µg·mL^{-1})	10 – 25	25
[DAb] (Dilution Factor)	100 – 500	250
BSA addition to DAb (%)	0.0 – 1.0	1.0
BSA addition to STR-ALP (%)	0.1 – 1.0	0.1
t (Ara h 1 & Ara h 6) (min)	30 – 60	60
t (DAb) (min)	30 – 60	30
Temperature (ºC)	25 – 30	30

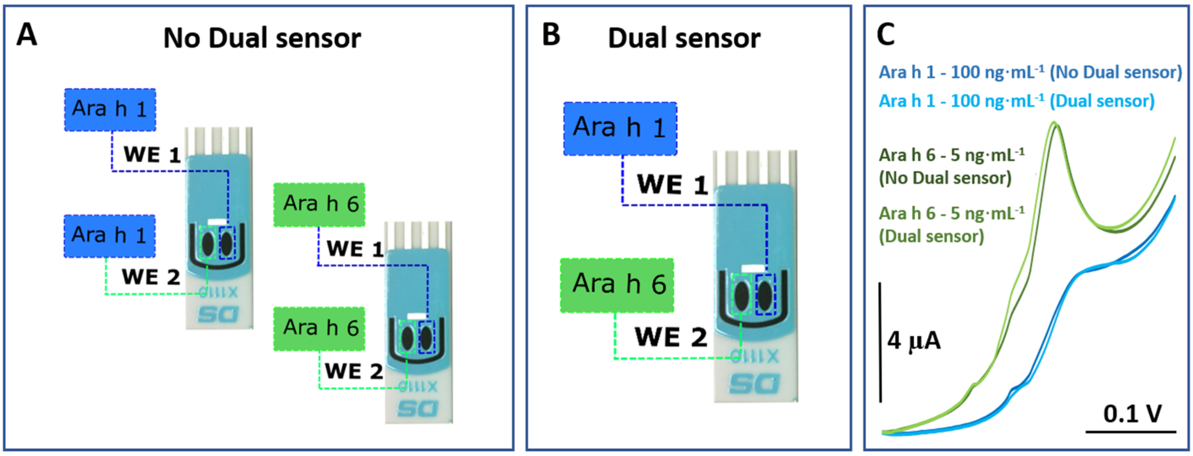

Figure S1. Schematic representation of the detection of Ara h 1 and Ara h 6 using a **(A)** No Dual sensor and **(B)** Dual sensor. **(C)** LSV voltammograms of the 'No Dual' and the Dual sensor (Ara h 1: 100·ng mL^{-1} and Ara h 6: 5 ng·mL^{-1}).

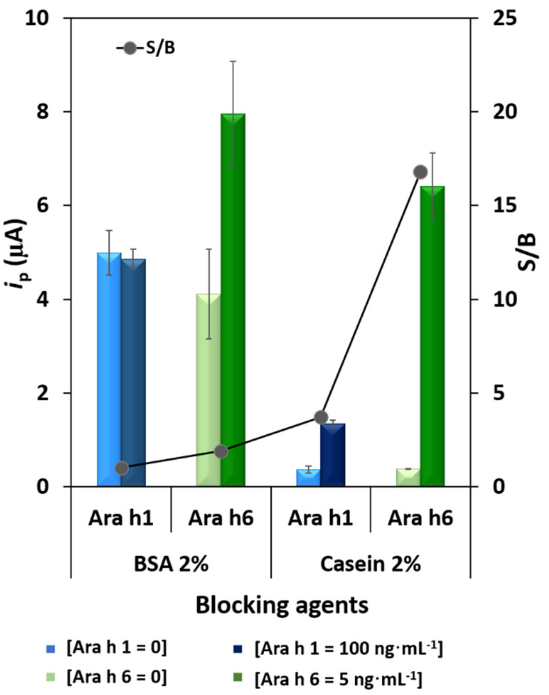

Figure S2. Study of the blocking agents BSA (2% (w/v)) and casein (2% (w/v)) and evaluation of non-specific adsorptions. (Ara h 1: 0 (blank) and 100 ng·mL^{-1}; Ara h 6: 0 (blank) and 5 ng·mL^{-1}; error bars correspond to the standard deviation of 3 replicates).

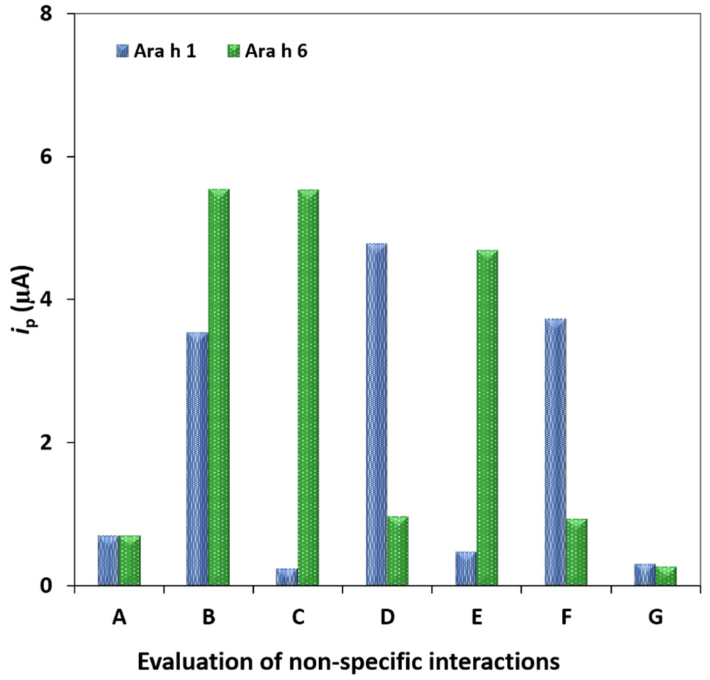

Figure S3. Effect of non-specific adsorptions of the target proteins and the capture and detection antibodies. (**A**) Blank and (**B**) 250 ng·mL^{-1} Ara h 1 and 2.5 ng·mL^{-1} Ara h 6 (**control assays**), Assays without the use of: Ara h 1 CAb (**C**), Ara h 6 CAb (**D**), Ara h 1 DAb (**E**), Ara h 6 DAb (**F**), Ara h 1 DAb and Ara h 6 DAb (**G**). (Ara h 1: 250 ng mL^{-1}; Ara h 6: 2.5 ng·mL^{-1}).

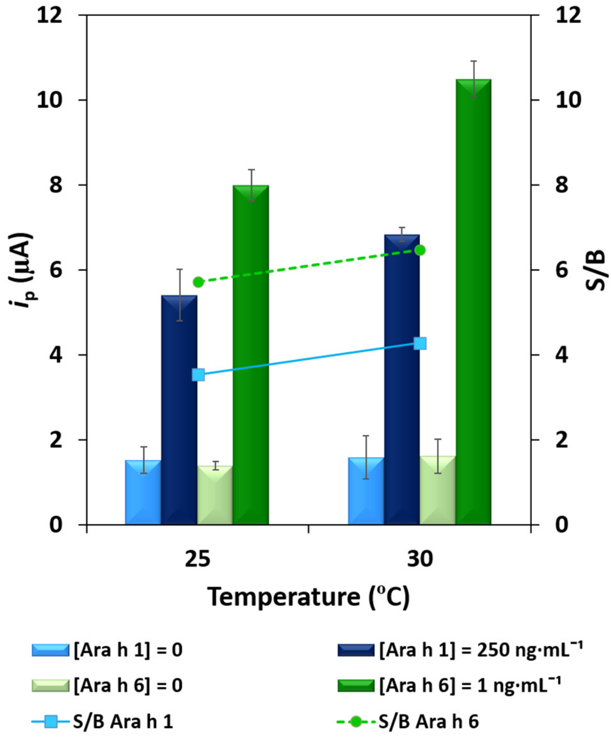

Figure S4. Current intensities obtained at 25 ºC and 30 ºC. (Ara h 1: 0 (blank) and 250 ng·mL^{-1}; Ara h 6: 0 (blank) and 1 ng·mL^{-1}; error bars correspond to the standard deviation of 3 replicates).

Table S2. Figures of merit of the immunoassay

Figure of merit	Ara h 1	Ara h 6
Concentration interval (ng·mL^{-1})	25 - 1000	0.050 - 1.0
Correlation coefficient (r)	0.997	0.998
Slope (m) (µA mL ng^{-1})	0.0111	7.10
Standard deviation of the slope (S_m) (µA mL ng^{-1})	0.0004	0.28
Intercept (b) (µA)	0.72	0.28
Standard deviation of the intercept (S_a) (µA)	0.21	0.17
Standard deviation of the method (S_{x0})	33.7	0.032
Coefficient of variation of the method (V_{x0}) (%)	10.5	3.31
Limit of detection (LOD) (ng·mL^{-1})	5.2	0.017
Limit of quantification (LOQ) (ng·mL^{-1})	17	0.055

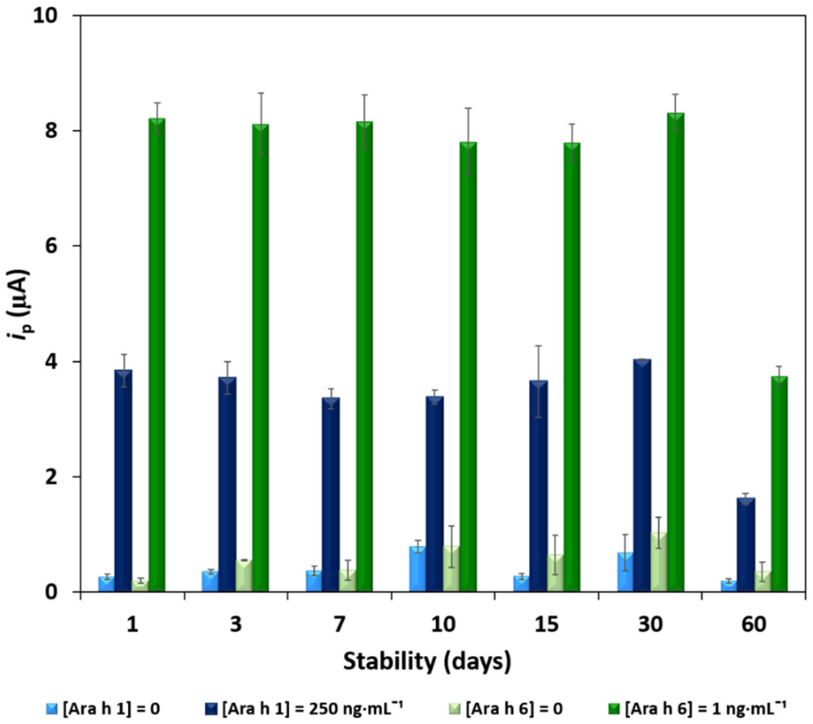

Figure S5. Storage stability of the immunosensor (Ara h 1 (0 (blank) and 250 ng·mL^{-1}) and Ara h 6 (0 (blank) and 1 ng·mL^{-1}); error bars correspond to the standard deviation of 3 replicates).

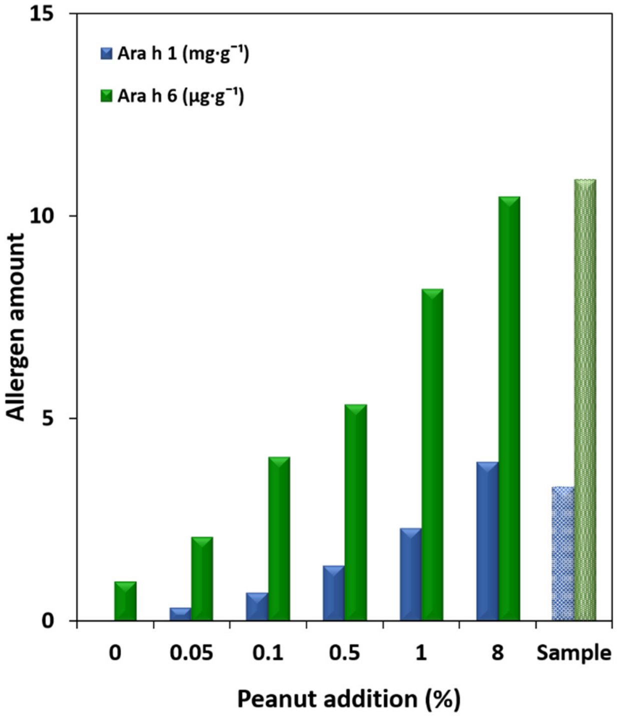

Figure S6. Quantification of Ara h 1 (mg·g^{-1}) and Ara h 6 (mg·g^{-1}) extracted from an allergen-free cookie sample spiked with increasing amounts of peanut and a peanut cookie.

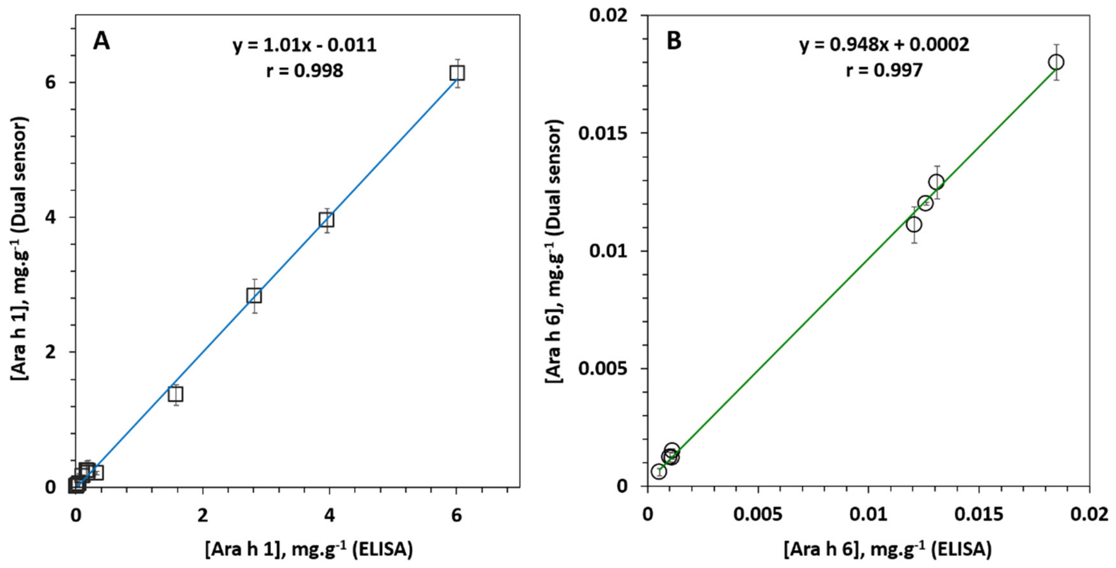

Figure S7. Correlation plots for the analysis of **(A)** Ara h 1 and **(B)** Ara h 6 in the tested food products using the developed immunosensor and ELISA kits. Error bars correspond to the standard deviation of 3 replicates.
